# Interrelationships of Economic Stressors, Mental Health Problems, Substance Use, and Intimate Partner Violence among Hispanic Emergency Department Patients: The Role of Language-Based Acculturation

**DOI:** 10.3390/ijerph182212230

**Published:** 2021-11-21

**Authors:** Carol B. Cunradi, Raul Caetano, William R. Ponicki, Harrison J. Alter

**Affiliations:** 1 Prevention Research Center, Pacific Institute for Research and Evaluation, 2150 Shattuck Avenue, Suite 601, Berkeley, CA 94704, USA; raul.caetano@utsouthwestern.edu (R.C.); bponicki@prev.org (W.R.P.); 2 Andrew Levitt Center for Social Emergency Medicine, Oakland, CA 94602, USA; harrison_alter@levittcenter.org

**Keywords:** Hispanic health paradox, language-based acculturation, emergency department, mental health, substance use, economic stressors

## Abstract

We analyzed the interrelationships of economic stressors, mental health problems, substance use, and intimate partner violence (IPV) among a sample of Hispanic emergency department patients and probed if Spanish language preference, which may represent low acculturation and/or immigrant status, had a protective effect, in accordance with the Hispanic health paradox. Study participants (*n* = 520; 50% female; 71% Spanish speakers) provided cross-sectional survey data. Gender-stratified logistic regression models were estimated for mental health problems (PTSD, anxiety, depression), substance use (risky drinking, cannabis, illicit drug use), and IPV. Results showed that economic stressors were linked with mental health problems among men and women. Among men, PTSD was associated with greater odds of cannabis and illicit drug use. Men who used cannabis and illicit drugs were more likely to report IPV. Male Spanish speakers had lower odds of anxiety and cannabis use than English speakers. Female Spanish speakers had lower odds of substance use and IPV than English speakers. The protective effect of Spanish language preference on some mental health, substance use, and IPV outcomes was more pronounced among women. Future research should identify the mechanisms that underlie the protective effect of Spanish language preference and explore factors that contribute to the observed gender differences.

## 1. Introduction

Hispanics constitute the largest ethnic minority group within the U.S., with 60.6 million individuals, representing 18.5% of the U.S. population [1]. The term Hispanic typically refers to individuals who have migrated to the U.S. from Central and South American countries and Spain, or their ancestors if they have lived in the U.S. for generations, and who identify as Hispanic and share a language, religion, and other cultural characteristics [2]. Mexicans account for the largest subgroup of Hispanics; other significant subpopulations include Cuban, Puerto Rican, Central American and Caribbean, and South American people [2]. Compared to non-Hispanic whites/Caucasians, Hispanics have poorer indicators of socioeconomic status (SES). For example, the poverty rate in 2019 for Hispanics was 15.7%, more than double the rate for non-Hispanic whites/Caucasians (7.3%) [3]. During the second quarter of 2020 (the outset of the COVID-19 pandemic), unemployment rates among Hispanics and non-Hispanic whites, respectively, were 16.7% and 12.0% [4]. Although unemployment rates have declined since the height of the pandemic, the disparities in rate persist [4]. In terms of education, Hispanics have the highest percentage among the major U.S. racial/ethnic groups of those age 25 or older who lack a high school diploma, a disparity that is greatest among Hispanics not born in the U.S. [5]. Finally, as of 2019 the percentage of those under 65 who remain uninsured is higher among Hispanics (19%) than any other racial/ethnic group, except Native Americans/Alaska Natives (21.8%); among non-Hispanic whites/Caucasians, 7.5% are uninsured [6]. Furthermore, among uninsured Hispanics, 33% are ineligible for ACA (Affordable Care Act) insurance coverage, due to their immigration status [6].

### 1.1. Hispanic Health Paradox

Despite these socioeconomic inequities, a substantial body of research has found that immigrant Hispanics have better health indicators [2,7,8,9] and a mortality advantage [10] compared to U.S.-born Hispanics and non-Hispanic whites/Caucasians. Often referred to as the Hispanic or immigrant health paradox [11,12], health advantages have been observed for physical health outcomes, such as perinatal outcomes (pre-term birth, low birth weight, and infant mortality) [7], coronary heart disease and stroke [13], and mental health outcomes [14], including substance use disorders [15]. Acculturation, generally described as ‘the processes whereby immigrants change their behavior and attitudes toward those of the host society’ [16], has been proposed as a sociocultural mechanism by which Hispanic immigrants lose their health advantage over U.S.-born Hispanics, as they navigate between prevailing American and traditional Latino cultures, and contend with adopting Euro-American behavioral practices, values, and cultural identities [17]. Levels of acculturation can be measured with multi-item scales, for example in [18,19,20,21]; another common approach is to use language as a proxy measure of acculturation [22]. Language is also correlated with other proxy measures, such as nativity, time in the U.S., and generational status [23].

### 1.2. Psychiatric Outcomes

Examples of the Hispanic health paradox can be seen for psychiatric outcomes. For instance, an analysis of data from the National Epidemiologic Survey on Alcohol and Related Conditions (NESARC) found that foreign-born Mexican Americans and foreign-born non-Hispanic whites had lower odds of DSM-IV substance use and mood and anxiety disorders compared with U.S.-born Hispanics and non-Hispanic whites [15]. Moreover, U.S.-born Mexican Americans had a lower risk of psychiatric morbidity compared to U.S.-born non-Hispanic whites [15]. Similarly, in a nationally representative sample from the National Comorbidity Survey Replication (NCS-R), Mexican immigrants had a lower lifetime risk of anxiety, mood, impulse control, and substance use disorders than U.S-born Hispanics and non-Hispanics [14]. Interestingly, the researchers found that those who immigrated to the U.S. at age 12 or younger had the same risk as non-immigrants for mood and impulse control disorders [14]. An analysis of a nationally representative sample of Hispanics from the National Latino and Asian American Study (NLAAS) found that the prevalence of psychiatric disorders varied among ethnic subgroups, with Puerto Ricans having the highest overall prevalence rate [24]. Other factors associated with elevated rates of psychiatric disorders were being U.S.-born, English-language-proficient, and third-generation Hispanic [24].

### 1.3. Intimate Partner Violence

In terms of intimate partner violence (IPV), a recent review found that Hispanic women who were U.S.-born reported greater prevalence rates of victimization, compared to those not born in the U.S. [25]. In an analysis of Hispanic men who participated in the NESARC study (Wave II), no differences in rates of past-year IPV perpetration were found between U.S.-born and immigrant men [26]. Among this sample, however, the U.S.-born men reported greater anxiety symptoms, alcohol dependence symptoms, and drug dependence symptoms than the immigrant men [26]. In a study among all married/partnered NESARC participants, Hispanics had higher IPV prevalence (victimization, perpetration, and bidirectional IPV) than whites, but lower prevalence than Blacks/African Americans [27]. Acculturation-related factors, such as nativity and language preference, were not included in the analysis [27]. An analysis of a national sample of married or cohabiting Hispanic men and women who participated the National Household Survey on Drug Abuse found no significant differences in the proportion of men and women reporting IPV perpetration (6.1% vs. 6.5%) or IPV victimization (8.8% vs. 7.8%) [28]. The results of the study’s gender-stratified multivariate analyses did not show an association between acculturation-related factors (survey language; nativity) and past-year IPV perpetration or victimization [28].

### 1.4. Study Goals and Significance

The purpose of this study is to analyze the interrelationships of economic stressors, mental health problems, substance use, and IPV among a sample of Hispanic ED patients, and to assess if Spanish language preference has a protective effect. Assessing these outcomes among Hispanics in an urban ED setting is significant for several reasons. For example, the emergency department (ED) increasingly serves as a safety net for underserved patients to receive medical care, especially those on Medicaid [29]. Compared to whites/Caucasians, Blacks and Hispanics are more likely to report receiving routine healthcare in an ED and are less likely to report having a primary care provider [30,31,32]. Urban ED patients comprise an at-risk, socially disadvantaged population, with elevated rates of unemployment, substance use, and mental health problems [33,34]. A recent ED-based study found that 37% of participants screened positively for social risks, defined as adverse social conditions associated with poor health, such as housing instability and food insecurity [35]. Additionally, compared to adults in the general household population, ED patients have an elevated prevalence of IPV. For example, rates of past-year IPV among male and female patients screened in ED-based studies range from 8.7% to 37% [36]. In contrast, the results of representative U.S. household population surveys indicate that between 4–7% of adults reported past-year IPV involvement [37]. Given the essential function of urban EDs in providing care to socially disadvantaged patients, including racial/ethnic minorities, the results of the current study can provide insights into how economic stressors may be associated with mental health problems, and thereafter substance use and IPV among Hispanic patients, as well as if those with a Spanish language preference have lower odds of these outcomes than those with an English language preference. The results can inform future research studies and interventions aimed at reducing health disparities among low SES Hispanic populations.

Our study hypotheses are as follows:

**Hypothesis** **1** **(H1).** *Economic stressors will be positively associated with mental health problems (PTSD, anxiety, depression)*.

**Hypothesis** **2** **(H2).** *Mental health problems will be positively associated with substance use (risky drinking, cannabis, illicit drug use)*.

**Hypothesis** **3** **(H3).** *Substance use will be positively associated with IPV perpetration and victimization*.

**Hypothesis** **4** **(H4).** *Spanish speaking study participants will have lower odds of mental health problems, substance use, and IPV perpetration and victimization than English speaking participants*.

## 2. Materials and Methods

### 2.1. Sample and Data Collection

Data for this cross-sectional study on drinking and IPV were obtained at the ED of an urban Level I trauma center in Northern California. As part of a county-wide integrated public health care system, the hospital’s ED provides care for the poor and uninsured. Patients seeking non-emergency care at the ED were considered eligible for the study if they met the following eligibility criteria: 18–50 years old; English or Spanish speaker; resident of the county in which the hospital is located; and married, cohabiting, or in a romantic (dating) relationship for the past 12 months. Exclusion criteria were patients who were intoxicated, experiencing acute psychosis or suicidal or homicidal ideation; cognitively/psychologically impaired and unable to provide informed consent; held in custody by law enforcement; or in need of immediate medical attention. The Alameda Health System Institutional Review Board approved the project’s protocol for the protection of human subjects.

The research staff conducted survey interviews with 1037 participants (53% female) between February and December 2017. Data collection procedures have been described elsewhere [38]. The study participant recruitment sequence is illustrated in Figure S1. As a first step, trained research assistants (RAs) identified potentially eligible participants through the ED’s electronic patient information system. They conducted face-to-face screening in the ED waiting room or in a treatment cubicle. If a patient screened eligible, the RA conducted the survey interview in the patient’s room, without others present, using computer-assisted personal interview (CAPI) techniques. Before being interviewed, participants provided informed consent. Recruitment of the sample occurred during weekdays from 9 a.m. to 9 p.m. Patients could opt to be interviewed in English or Spanish. Participants who chose to be interviewed in Spanish were interviewed by bilingual, Spanish-speaking RAs. The Spanish version of the questionnaire had been validated through translation into Spanish and retranslation into English, followed by verification. Average survey interview completion time was 37 min (SD = 20.7). Participants received a USD 30 grocery store gift card as an incentive for completing the survey. The current analysis is limited to those participants who self-reported their race/ethnicity in the survey as Hispanic or Latino (*n* = 520).

### 2.2. Measurements

Gender. Self-reported gender was coded as male or female.

Age. This was used as a continuous variable.

Household food insufficiency. Participants rated their level of agreement with the statement, ‘In the past 12 months, the food we bought ran out and we didn’t have money to get more’ (never; sometimes true; often true). We dichotomized and compared those who responded ‘sometimes’ or ‘often’ to those who responded ‘never’ [39]. Those in the latter category comprised the reference group.

Household financial strain. Participants were asked, ‘How would you describe the money situation in your household right now?’ Response options were: (1) comfortable with extra; (2) enough but no extra; (3) have to cut back; (4) cannot make ends meet. We dichotomized and compared those who chose responses 3–4 to those who chose responses 1–2 [39]. Those in the latter category comprised the reference group.

Fired/laid off from job. Participants were asked if in the past 12 months they had been fired or laid off from a job. We dichotomized and compared those who responded ‘yes’ to those who responded ‘no’. Those in the latter category comprised the reference group.

Anxiety. This was measured with 7 items on a 4-point Likert-type scale, from the Hospital Anxiety and Depression scale [40]. A cutoff point equal to or greater than 8 identified those positive for anxiety. The reference group were those who scored less than 8. Cronbach’s α was 0.81.

Depression. This was measured with 7 items on a 4-point Likert-type scale from the Hospital Anxiety and Depression Scale [40]. A cutoff point of 8 or more identified those positive for depression. The reference group were those who scored less than 8. Cronbach’s α was 0.69 for depression.

Post-Traumatic Stress Disorder (PTSD). We measured PTSD with the 4-item Primary Care Screener for PTSD [41]. A score of 3 or more was considered positive. A dichotomous variable was created for those who scored positively; the reference group were those who did not have a positive score. Cronbach’s α was 0.83.

At-risk drinking. We asked participants who drank alcohol in the past 4 weeks, ‘What was the greatest number of drinks you had on any day in the past 4 weeks?’. A ‘drink’ was defined as a 12-ounce can of beer, a five-ounce glass of wine, or a one-ounce shot of liquor. We asked participants who did not use alcohol in the past 4 weeks the same question over the past year. Women/men were considered at-risk drinkers if they had had four/five or more drinks on any one day in the past 4 weeks (past 12 months for past year drinkers) [42]. For women, the reference group was those who had less than four drinks on any one day; for men, the reference group were those who had less than five drinks in one day.

Cannabis use. We asked participants, ‘How many times during the past 12 months, or 365 days, did you use marijuana or hashish (weed, pot, hash) without a doctor’s instruction?’. We created a dichotomous variable representing any past-year cannabis use. The reference group were those who did not report any past-year cannabis use. Recreational cannabis use was legalized in California for those 21+ years old in November 2016.

Illicit drug use. We asked participants, ‘How many times during the past 12 months, or 365 days, did you use amphetamines, cocaine, heroin, and pain relievers not prescribed for you?’. We created a dichotomous variable representing any past-year illicit drug use. The reference group were those who did not report any past-year illicit drug use.

Intimate partner violence: We measured past-12-month physical IPV victimization and perpetration with the 12-item physical assault subscale of the CTS2, Revised Conflict Tactics Scale [43]. Participants were asked how many times in the past 12 months their spouse or partner did each type of aggressive behavior to them, and how many times they did each physically aggressive behavior to their spouse or partner. Separate dichotomous variables were created for any IPV perpetration and any IPV victimization. The reference groups were those who did not report any past 12-month IPV perpetration or IPV victimization, respectively. Cronbach’s α for the scale in the dataset under analysis was 0.85.

### 2.3. Statistical Analysis

We conducted data analyses using IBM SPSS v. 25 (IBM, Armonk, NY, USA). We used Fisher’s exact test to analyze gender differences in categorical participant characteristics. An independent-samples t-test was used to analyze gender differences in mean age. We then estimated a series of gender-stratified multivariate logistic regression models for three mental health outcomes (PTSD, anxiety, depression), three substance use outcomes (at-risk drinking, cannabis use, illicit drug use) and two IPV outcomes (IPV perpetration, IPV victimization). The models generated adjusted odds ratios (AORs) and 95% confidence intervals (CIs) that quantified the strength of the association between the independent (exposure) variable and the outcome (dependent variable) after accounting for the other variables in the model. We stratified the models by gender, due to previous results showing significant gender differences in drinking, drug use, and IPV [44,45], and prior studies showing a more robust association between acculturation and alcohol use outcomes among Hispanic women than men [17]. In accordance with H1, we analyzed the association between economic stressors (being fired/laid off from a job, food insufficiency, financial strain) and each mental health outcome. For H2, we analyzed the association between PTSD, anxiety, and depression and each substance use outcome. For H3, we analyzed the association between at-risk drinking, cannabis, and illicit drug use and each IPV outcome. To test H4, we included a language preference variable in each model. All models were adjusted for age. Missing data for the variables in the study were minimal (range 0–1.6%); these were removed through list-wise deletion from the analysis.

## 3. Results

### 3.1. Sample Characteristics

The sample characteristics are shown in Table 1. Most participants (>70%) chose to have their survey interview conducted in Spanish. Substantial proportions of the sample reported economic stressors. For example, more than one third of men reported having been fired or laid off from a job in the past year, as did over one quarter of women. Approximately half the sample reported food insufficiency. More than 40% of men and nearly half of women reported financial strain. A greater proportion of women than men screened positively for depression (19.5% vs. 12.1%; *p* = 0.02). In terms of substance use, there were significant gender differences in at-risk drinking (38.7% of men vs 14.1% of women; *p* < 0.001) and illicit drug use (12.6% of men vs 4.3% of women; *p* = 0.001). A similar proportion of men and women reported past-year IPV perpetration.

### 3.2. Multivariate Results

#### 3.2.1. Economic Stressors and Mental Health Problems

Associations between economic stressors and mental health problems are shown in Table 2. Among men, the results of the adjusted models show that food insufficiency was associated with a more than four-fold increase in the likelihood of PTSD, anxiety, and depression. In addition, men who had been fired or laid off from their job were more than twice as likely to screen positively for anxiety. Male Spanish speakers had lower odds of anxiety compared to English speakers (AOR = 0.42; 95% CI 0.20, 0.90; *p* < 0.05). Among women, results of the adjusted models showed that food insufficiency was associated with a five-fold increase in the likelihood of PTSD. Financial strain was associated with more than double the odds of anxiety. None of the economic stressors were significantly associated with depression, nor were Spanish speakers less likely to have mental health problems than English speakers.

#### 3.2.2. Mental Health Problems and Substance Use

Associations between mental health problems and substance use outcomes are shown in Table 3. Among men, the results of the adjusted models showed that those who screened positively for PTSD had a more than three-fold increased likelihood of cannabis use, and were more than twice as likely to use illicit drugs. Male Spanish speakers had decreased odds of cannabis use compared to English speakers (AOR = 0.22; 95% CI 0.20, 0.90; *p* < 0.01). Men’s mental health problems were not associated with increased odds of risky drinking. Among women, the results of the adjusted models showed that mental health problems were not associated with any of the substance use outcomes. Female Spanish speakers had lower odds of risky drinking (AOR = 0.31; 95% CI 0.15, 0.66; *p* < 0.01), cannabis use (AOR = 0.08; 95% CI 0.03, 0.25; *p* < 0.001), and illicit drug use (AOR = 0.21; 95% CI 0.06, 0.80; *p* < 0.05) compared to English speakers. 

#### 3.2.3. Substance Use and Intimate Partner Violence

Associations between substance use and IPV outcomes are shown in Table 4. Among men, the results of the adjusted models showed that cannabis use was associated with a four-fold increased risk of IPV perpetration, and illicit drug use was associated with a more than three-fold increased likelihood of this outcome. Men who used illicit drugs had four-fold elevated odds of IPV victimization. Among women, the results of the adjusted models showed that substance use was not associated with IPV perpetration or victimization. Women Spanish speakers had reduced odds of IPV perpetration compared to English speakers (AOR = 0.30; 95% CI 0.11, 0.82; *p* < 0.05); there was no protective effect for IPV victimization.

## 4. Discussion

Among a socially disadvantaged sample of Hispanic men and women seeking medical care at an urban safety-net hospital ED, a substantial proportion reported economic stressors and screened positively for mental health problems. For example, regarding economic stressors, more than one quarter of the women and nearly 40% of the men reported having been fired or laid off from their job. Nearly half of respondents reported food insufficiency; over 40% of men and nearly 50% of women reported financial strain. In terms of mental health, anxiety was the most prevalent problem reported by men and women. While men had greater rates of risky drinking and illicit drug use than women, no gender differences were seen for self-reported IPV perpetration or victimization. Previous analyses of the data showed that Hispanic study participants had lower rates of past-year substance use and IPV compared to study participants who self-reported their race/ethnicity as white, Black/African American, or multiethnic/multiracial [38].

### 4.1. Impact of Economic Stressors on Mental Health 

The H1 hypothesized associations between economic stressors and mental health problems were partially confirmed, with some gender differences. Specifically, food insufficiency was associated with PTSD, anxiety, and depression among men; among women, food insufficiency was only associated with PTSD. Being fired/laid off from a job was only associated with anxiety among men; financial strain was only associated with anxiety among women. The results suggest that Hispanic ED patients who experience economic stressors are significantly more likely to screen positively for mental health problems than those who are not exposed to these stressors. In contrast with past research [46], economic stressors were not associated with depression among women. One explanation is that the impact of economic stressors may be mitigated by other sample characteristics. For example, family separation is associated with depression among Mexican-origin women residing in the U.S. [46]; however, the current sample consists entirely of married or partnered participants. The women’s married/partnered status may, therefore, help buffer the influence of economic stressors on depression.

### 4.2. Men’s, Not Women’s, Mental Health Problems Linked to Substance Use

The results showed that the H2 hypothesized associations between mental health problems and substance use were not confirmed among women. Among men, no associations were observed between mental health problems and odds for risky drinking. Men who screened positively for PTSD, however, had elevated odds of cannabis use and illicit drug use, compared to men who did not screen positively for PTSD. Men’s anxiety and depression were not associated with elevated odds of cannabis or illicit drug use. Other factors besides mental health problems may be more salient correlates of substance use among socially disadvantaged Hispanics. For example, a longitudinal study among Hispanic young adults found that exposure to adverse childhood experiences was associated with binge drinking and cannabis use, even after accounting for culturally relevant variables [47]. Other potential correlates of substance use outcomes among Hispanic populations are impulsivity traits [48] and emotion dysregulation [49].

### 4.3. Men’s, Not Women’s, Cannabis and Drug Use Associated with IPV

The H3 hypothesized association between substance use and IPV was partially confirmed, but only among men. No association was observed between women’s substance use and IPV perpetration or victimization. It may be that each partner’s psychosocial traits, such as level of impulsivity, are more likely to contribute to the expression of aggressive dyadic behavior among Hispanic couples [50]. Among men, cannabis and illicit drug use were associated with IPV perpetration; illicit drug use was also associated with IPV victimization. The lack of an observed association between risky drinking and IPV perpetration or victimization is consistent with the findings reported by Caetano and colleagues [51]. In their longitudinal follow-up study, which accounted for acculturation and acculturation stress among Hispanic couples, binge drinking by either partner did not predict IPV perpetration or victimization [51]. 

### 4.4. Protective Effect of Spanish-Language Preference: Gender Differences

The H4 hypothesized protective effects of Spanish language preference on mental health problems, substance use, and IPV were partially confirmed, with notable gender differences. For example, in terms of mental health problems, Spanish language preference was associated with lower odds of anxiety among men; no protective effect was observed for women. On the other hand, Spanish language preference was associated with lower odds for risky drinking, cannabis use, and illicit drug use among women; for men, a protective effect was only seen for cannabis use. The gender differences in the association between Spanish language preference and risky drinking are consistent with those described in two previous reviews, both of which noted more robust findings for this association among women [17,52]. The consistent protective effect of Spanish language preference among women may be attributed to sharper differences in gender roles in Latino cultures compared to Anglo culture. In the former, the consequences of behavioral problems such as risky drinking and drug use are more severe for women than for men. Societal drinking norms are stricter for women than men. Spanish language preference may be a marker for women that adhere to these stricter gender-related norms. Finally, Spanish language preference was associated with lower odds of IPV perpetration among women, but not men. The protective effect of Spanish language preference may be due to Latino cultural factors centered around family life, or perhaps the positive side of machismo where men are ‘protectors’ of the family. It is also possible that the group with language preference for Spanish is a selected special group, with more people born abroad and therefore more immigrants who could be more resilient than others (i.e., self-selection). The acculturation process may also result in dyadic stress that increases the likelihood of IPV. For example, an analysis of a national sample of Hispanic couples found that compared to couples in which both partners were categorized as low acculturation, based on a multi-item scale, couples in which both partners were categorized as medium acculturation had the highest odds for female-to-male partner violence [50]. Similarly, in a longitudinal follow-up, lower acculturation among the men was associated with higher acculturation stress, which was directly related to greater likelihood of IPV. The same associations were observed among women; additionally, higher levels of acculturation among women were directly linked to IPV [51].

### 4.5. Study Strengths and Limitations

This study is characterized by various strengths. For example, the study is among the first to examine the interrelationships of economic stressors, mental health problems, substance use, and IPV among a sample of Hispanic ED patients, and to assess if Spanish language preference has a protective effect. The persistence of socioeconomic disparities among the U.S. Hispanic population underscores the importance of identifying how economic stressors are associated with mental health problems, and how mental health problems are related to substance use and IPV. Another study strength is the near-equal number of men and women study participants. This allowed for a gender-stratified analysis, to compare the prevalence and correlates of the study outcomes. Since IPV is most often bidirectional (i.e., men and women report both perpetration and victimization) [27,28,53,54,55], it is important to measure both behaviors independently. By measuring IPV perpetration and victimization among all study participants, this study helps to overcome a limitation of some previous IPV research among Hispanics, in which factors related to IPV perpetration were confined to male study participants [26,56,57], and factors related to IPV victimization were confined to female study participants [58,59,60,61].

There are several study limitations that should be considered. First, due to the cross-sectional study design, causality cannot be inferred. It is also important to note that many of the associations observed in this study could be bidirectional. For example, risky drinking may increase the odds of experiencing IPV, but experiencing IPV could lead to increased substance use [62]. Similarly, our models tested the association between economic stressors and mental health problems; we did not test the association between IPV exposure and mental health problems. Prior research has shown that IPV victimization is associated with elevated odds for PTSD, anxiety, and depression [63]. Second, because the sample was obtained from a single safety-net ED, the generalizability of the findings may be limited. Third, the survey did not assess acculturation stress, nativity, or immigrant status, nor were multi-item measures of acculturation collected. We therefore largely refrain from using the term acculturation, as suggested by Doucerain et al. [64], using instead language preference, to identify the measurement more clearly in the analysis. Fourth, the study lacks concurrent dyadic reports on IPV, since the participants’ spouses and romantic partners were not interviewed. This may result in an underestimate of IPV [65]. Fifth, we note that due to the study’s exclusion criteria, the sample did not include those with suicidal ideation. As those with suicidal behavior exhibit elevated rates of substance use disorders, impulsivity, and aggressive behavior [66], inclusion of this group would likely have produced significantly different results than the current study findings. Finally, recall bias may have affected participants’ ability to recall past-year drinking, drug use, and other behaviors.

## 5. Conclusions

The results of this study suggest that socially disadvantaged Hispanics seeking non-acute care at urban EDs may be especially vulnerable to mental health problems. This has important implications for clinical providers in similar urban safety-net settings. For example, given the high levels of food insufficiency observed in this sample, ED staff should be aware that their patients may benefit from referrals to community resources, such as food banks, to bolster household nutrition. Similarly, ED staff may want to consider using brief screeners to assess for anxiety and other mental health problems among their patients. ED staff can provide brief counseling to those who screen positively and provide referrals to outpatient mental health services. A fundamental concern for ED staff should be ensuring that low English proficiency patients receive appropriate Spanish language assistance during their ED visit [67].

The study results indicate that Spanish language preference, which may represent low acculturation and/or immigrant status, was protective against men’s anxiety and cannabis use, and against women’s risky drinking, cannabis, and illicit drug use, and IPV perpetration. Future research should identify the mechanisms that underlie this protective effect and determine the factors that contribute to a greater protective effect among women. Obtaining dyadic reports using a longitudinal study design and using appropriate dyadic modeling techniques will enhance these goals.

## Figures and Tables

**Table 1:** Sample Characteristics.

Variable	Males (*n* = 256) % or Mean (SD)	Females (*n* = 262) % or Mean (SD)	*p* Value *
Age	35.89 (8.25)	34.88 (7.87)	0.16
Spanish survey	72.3	70.6	0.70
**Economic stressors**			
Fired/laid off from job	38.6	28.8	0.02
Food insufficiency	46.5	52.1	0.22
Financial strain	43.9	48.3	0.33
**Mental health problems**			
PTSD	15.6	22.1	0.07
Anxiety	25.0	31.4	0.12
Depression	12.1	19.5	0.02
**Substance use**			
At-risk drinking	38.7	14.1	<0.001
Cannabis use	12.3	8.6	0.19
Drug use	12.6	4.3	<0.001
**Intimate partner violence**			
IPV perpetration	9.9	9.6	1.00
IPV victimization	15.0	9.6	0.08

* Fisher’s exact test or independent-samples t-test for gender differences in sample characteristics.

**Table 2:** Associations between economic stressors and mental health problems.

	PTSD		Anxiety		Depression	
	Men	Women	Men	Women	Men	Women
	AOR (95% CI)	AOR (95% CI)	AOR (95% CI)	AOR (95% CI)	AOR (95% CI)	AOR (95% CI)
Age	0.93 (0.89, 0.98)	1.01 (0.96, 1.05)	1.02 (0.98, 1.06)	1.02 (0.99, 1.06)	0.99 (0.94, 1.04)	1.02 (0.99, 1.07)
Spanish lang. survey	2.23 (0.87, 5.69)	0.94 (0.47, 1.86)	0.42 (0.20, 0.90) ^a^	0.77 (0.41, 1.43)	0.56 (0.22, 1.44)	0.99 (0.48, 2.03)
Fired/laid off from job	1.68 (0.81, 3.49)	0.71 (0.35, 1.42)	2.17 (1.17, 4.04) ^a^	1.42 (0.77, 2.61)	1.20 (0.54, 2.66)	1.00 (0.50, 2.02)
Food insufficiency	4.59 (2.03, 10.39) ^c^	5.09 (2.32, 11.19) ^c^	4.29 (2.21, 8.28) ^c^	1.61 (0.85, 3.03)	4.33 (1.74, 10.76) ^b^	1.94 (0.91, 4.13)
Financial strain	0.52 (0.23, 1.18)	0.96 (0.47, 1.95)	1.38 (0.70, 2.74)	2.67 (1.42, 4.99) ^b^	1.44 (0.60, 3.45)	1.97 (0.94, 4.14)

^a^*p* ≤ 0.05; ^b^ *p* ≤ 0.01; ^c^ *p* ≤ 0.001.

**Table 3:** Associations between mental health problems and substance use.

	Risky Drinking		Cannabis Use		Illicit Drug Use	
	Men	Women	Men	Women	Men	Women
	AOR (95% CI)	AOR (95% CI)	AOR (95% CI)	AOR (95% CI)	AOR (95% CI)	AOR (95% CI)
Age	1.01 (0.99, 1.05)	1.01 (0.97, 1.06)	0.93 (0.88, 0.99) ^a^	0.98 (0.92, 1.05)	0.95 (0.91, 1.00)	1.01 (0.99, 1.07)
Spanish lang. survey	0.71 (0.39, 1.30)	0.31 (0.15, 0.66) ^b^	0.22 (0.20, 0.90) ^b^	0.08 (0.03, 0.25) ^c^	0.60 (0.25, 1.42)	0.21 (0.06, 0.80) ^a^
PTSD	1.10 (0.51, 2.36)	1.03 (0.43, 2.47)	3.31 (1.18, 9.33) ^a^	1.11 (0.34, 3.61)	2.67 (1.03, 6.91) ^a^	0.78 (0.15, 4.09)
Anxiety	1.30 (0.68, 2.46)	1.08 (0.44, 2.66)	1.24 (0.47, 3.29)	1.00 (0.30, 3.32)	1.42 (0.57, 3.53)	1.16 (0.24, 5.56)
Depression	1.21 (0.55, 2.64)	1.08 (0.39, 2.975)	0.65 (0.70, 2.74)	0.36 (0.06, 2.04)	0.96 (0.31, 2.96)	0.36 (0.04, 3.72)

^a^*p* ≤ 0.05; ^b^ *p* = <0.01; ^c^ *p* ≤ 0.001.

**Table 4:** Associations between substance use and intimate partner violence.

	IPV Perpetration		IPV Victimization	
	Men	Women	Men	Women
	AOR (95% CI)	AOR (95% CI)	AOR (95% CI)	AOR (95% CI)
Age	0.98 (0.92, 1.05)	0.96 (0.91, 1.03)	0.96 (0.91, 1.01)	0.96 (0.90, 1.02)
Spanish lang. survey	0.63 (0.22, 1.76)	0.30 (0.11, 0.82) ^a^	0.48 (0.21, 1.11)	0.41 (0.16, 1.07)
Risky drinking	2.48 (0.97, 6.30)	2.30 (0.77, 6.85)	1.84 (0.85, 3.95)	2.25 (0.75, 6.74)
Cannabis use	4.02 (1.35, 11.95) ^a^	2.52 (0.73, 8.76)	1.13 (0.40, 3.20)	1.44 (0.39, 5.37)
Illicit drug use	3.32 (1.22, 9.05) ^a^	1.33 (0.26, 6.70)	4.30 (1.78, 10.39) ^b^	1.85 (0.38, 9.06)

^a^*p* ≤ 0.05; ^b^ *p* ≤ 0.01; ^c^ *p* ≤ 0.001.

## Data Availability

The data are not publicly available, due to Institutional Review Board restrictions.

## Supplementary Materials

The following are available online at https://www.mdpi.com/article/10.3390/ijerph182212230/s1, Figure S1: Study sample recruitment.
