# Supplementary figures and images for: Interrelationships of Economic Stressors, Mental Health Problems, Substance Use, and Intimate Partner Violence among Hispanic Emergency Department Patients: The Role of Language-Based Acculturation
Source: Int J Environ Res Public Health. 2021 Nov 21;18(22):12230. doi: 10.3390/ijerph182212230 (PMC8623040; doi:10.3390/ijerph182212230)

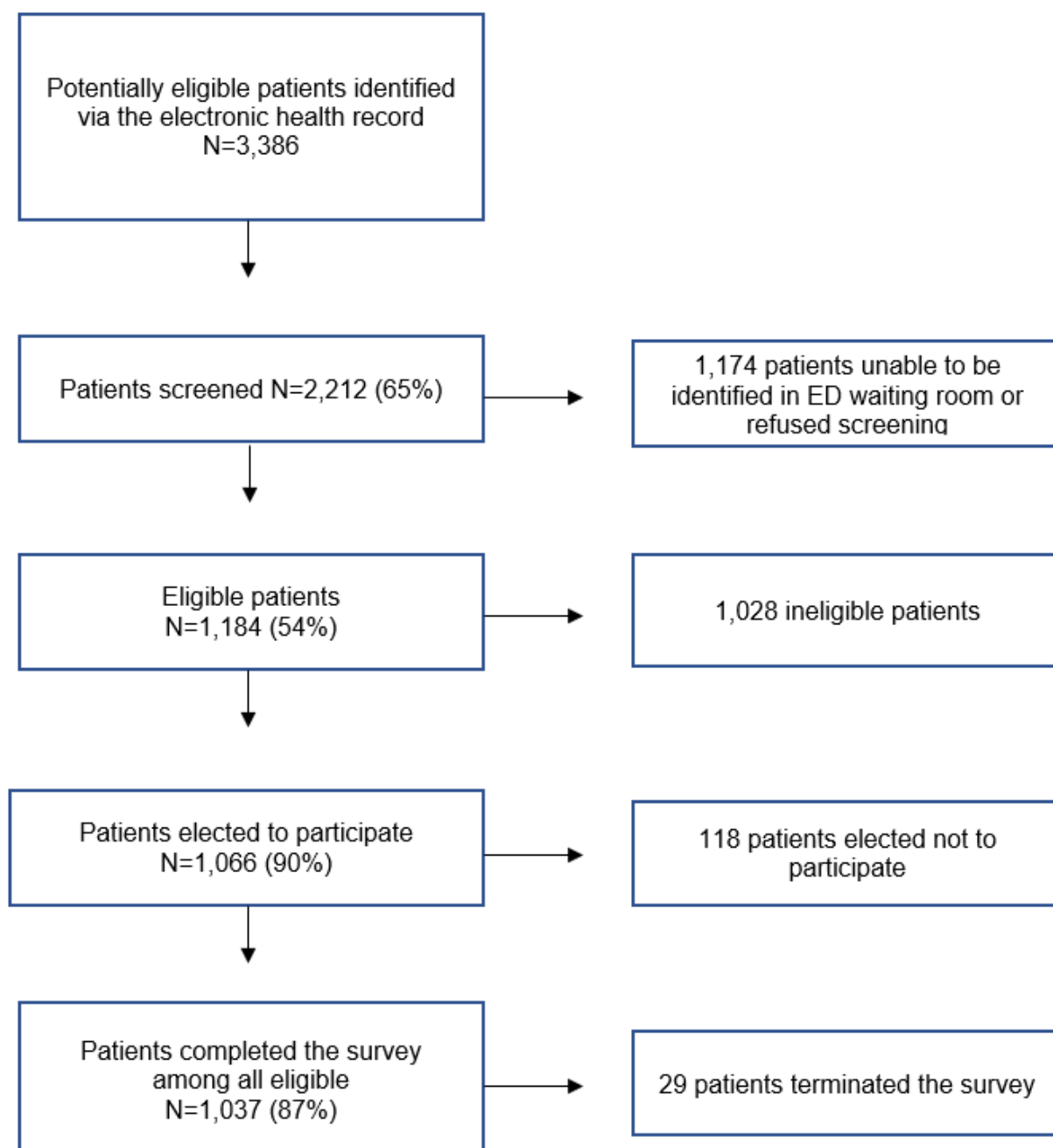

**Figure S1.** Study sample recruitment.

Supplement: Supplementary file 1 [file ijerph-18-12230-s001.zip › ijerph-1433194-supplementary.pdf]
